# Rare perivascular epithelial cell tumor of the colon ^18^F-FDG PET/CT imaging: A case report

**DOI:** 10.1097/MD.0000000000033802

**Published:** 2023-05-17

**Authors:** Qianqian Chen, Peiqi Wang, Xiao Zhang, Jinhe Zhang

**Affiliations:** a Guangzhou University of Chinese Medicine, Guangzhou, China; b Department of Nuclear Medicine, General Hospital of Southern Theater Command, Guangzhou, China.

**Keywords:** ^18^F-FDG PET/CT, colon, neoplasms with perivascular epithelioid cell differentiation

## Abstract

Neoplasms with perivascular epithelioid cell differentiation (PEComas) are mesenchymal tumors that rarely occur in the colon. Here, we report the occurrence of a malignant PEcoma in the colon using ^18^F-fluorodeoxyglucose positron emission tomography/computed tomography (^18^F-FDG PET/CT).

**Patient concerns:**

A 55-year-old woman was admitted to the hospital with abdominal pain for 10 days and a self-induced abdominal mass for 3 days. ^18^F-FDG PET/CT imaging showed a large hypermetabolic nodule and mass in the right mid-upper abdomen with heterogeneous density and a further increase in metabolism on the delayed scan.

**Diagnoses:**

PEComa of the colon.

**Interventions:**

Tumor resection was performed.

**Outcomes:**

The patient is well after 2 months of treatment, pending further follow-up.

**Lessons:**

Malignant perivascular epithelioid cell tumors originating in the colon are extremely rare, and our report suggests that PEComa should be considered as a differential diagnosis for ^18^F-FDG gastrointestinal malignancies. Additionally, ^18^F-FDG PET/CT may play a key role in the staging and extent of lesions in intestinal malignancies.

## 1. Introduction

The World Health Organization defines neoplasms with perivascular epithelioid cell differentiation (PEComa) as “a mesenchymal tumor comprising of histologically and immunohistochemically distinct perivascular epithelioid cells.” Typical PEComas include several solid types, such as vascular smooth muscle lipoma, lymphangioleiomyomatosis, and pulmonary clear cell “glycosarcoma.”^[1]^ To date, this sporadic perivascular epithelioid cell tumor has been reported to occur in different anatomical locations, such as the lungs, uterus, bone, retroperitoneum, ovaries, and liver.^[2–8]^ Most PEComas are benign, and malignant PEComas often show local recurrence, distant metastases, and poor prognosis.^[9,10]^ Here, we report a rare case of a malignant PEComa in the colon on ^18^F-fluorodeoxyglucose positron emission tomography/computed tomography (^18^F-FDG PET/CT).

## 2. Case report

A 55-year-old woman was admitted to the hospital with abdominal pain for 10 days and a self-induced abdominal mass for 3 days. The patient had abdominal pain, with no obvious cause 10 days prior, mainly in the right upper abdomen, with paroxysmal vague pain of variable duration, unrelated to body position and eating, with pain radiating to the lower back, accompanied by diarrhea and yellow dilute water, approximately 3 to 4 times/day. The patient abdomen was soft, with a slight bulge visible in the right upper abdomen. Abdominal breathing, no abdominal wall changes, and no intestinal pattern or intestinal peristaltic waves were observed. A tough mass of approximately 10 × 8 cm with fair borders was observed in the right upper middle abdomen, with mild localized pressure pain and no pressure or rebound pain. Laboratory tests showed glycogen 125:38.3 μ/mL, gamma-glutamyl transpeptidase 102 U/L, and alkaline phosphatase 223 U/L.

**Figure 1.**
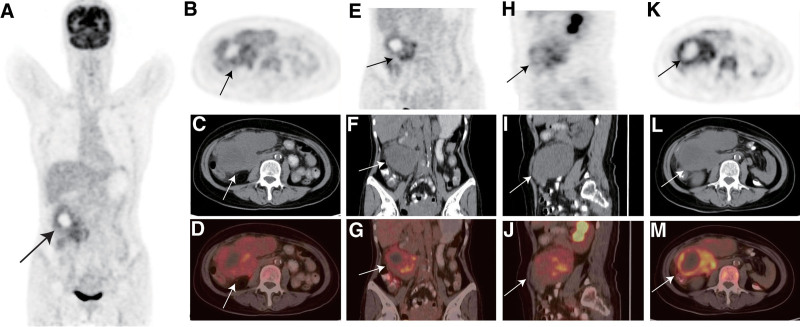
Maximum intensity projection image (A) showing a large irregular abnormal activity in the right abdomen (Black arrow). Axial (B: PET, C: CT, D: fused PET/CT), coronal (E: PET, F: CT, G: fused PET/CT) and sagittal (H: PET, I: CT, J: fused PET/CT) images show a right midupper abdominal mass measuring approximately 12.0 cm × 6.7 cm with a standardized uptake value maximum (SUVmax) of 5.4 on the first scan and a further increase in radioactive uptake with a SUVmax of 7.7 on the delayed scan (K: PET, L: CT, M: fused PET/CT). CT = computed tomography, PET = positron emission tomography, SUV = standardized uptake value.

The patient underwent a whole-body examination using ^18^F-fluorodeoxyglucose positron emission tomography/computed tomography (^18^F-FDG PET/CT) (Biograph 16, Siemens, Germany). PET/CT findings (Fig. 1): A large soft tissue mass with heterogeneous density was seen in the right upper abdomen, measuring approximately 12.0 cm × 6.7 cm, with increased radioactive uptake heterogeneity and a standardized uptake value maximum (SUVmax) of 5.4 on the first scan and 7.7 on the delayed scan. Hypodense foci were seen in the center of the mass with a computed tomography (CT) value of 21.5 Hu and inadequate radioactive uptake. The adjacent duodenum was compressed, the surrounding fat space was blurred, and several small lymph node shadows were observed around the lesion, with no increase in metabolism. PET/CT considers the disease to be a malignant tumor of mesenchymal tissue origin. The patient then underwent a tumor resection, PEComas were diagnosed in combination with hematoxylin-eosin staining and immunohistochemical markers.

## 3. Discussion

The main clinical manifestations of PEComas in the gastrointestinal tract are abdominal pain, black stools, rectal bleeding, obstruction, weight loss, anemia, and in some cases no symptoms, with abdominal pain being the most common clinical manifestation. Most PEComas appear as well-defined, uniformly dense masses on plain CT, which may have mixed internal density due to the presence of fat, necrotic cystic lesions, hemorrhage, and rarely, calcifications. In magnetic resonance imaging, lesions usually show low or isosignals on T1-weighted images and inhomogeneous high signals on T2-weighted images.^[11]^ CT and magnetic resonance imaging examinations are not sensitive enough for the diagnosis and differentiation of benign and malignant PEComas owing to their nonspecific imaging features but can help detect suspicious lymphovascular invasion and metastatic lesions. ^18^F-FDG PET/CT is valuable in differentiating malignant from benign PEComas and detecting metastases.^[12,13]^ FDG uptake values are usually not increased or are mildly increased in benign PEComas, whereas FDG uptake values are significantly increased in malignant PEComas and its metastases, with SUVmax up to 72.4.^[3]^ In this case, the main clinical manifestations were abdominal pain and weight loss. Abdominal pain may be caused by compression, embolism, or tumor bleeding. In this case, the lesion was mainly located in the right upper abdomen, poorly demarcated from the ascending colon, and the mass was growing outwards, and was large in size, with inhomogeneous density within it. Adjacent tissues were shifted by compression, and SUV uptake showed a heterogeneous increase, with SUVmax of 5.4 on the first scan of some lesions and a further increase of 7.7 on the delayed scan. This is consistent with the malignant tumor manifestations and pathological findings. A radiological defect at the center of the lesion might be associated with hemorrhagic necrosis.

In addition, this tumor should be differentiated from extragastric mesenchymal tumors, mesenteric lymphomas, peritoneal mesotheliomas, and other tumors on CT. Extragastric mesenchymal tumors are mostly solitary and large in size, most of which do not communicate with the intestine; the tumor border is clear and may be irregular in shape. Mesenteric lymphomas are primarily characterized by mesenteric lymph node involvement, with multiple enlarged mesenteric lymph nodes fusing to form irregularly shaped masses. Peritoneal mesotheliomas are diffuse in nature, showing irregular thickening of the peritoneum, greater omentum and mesentery in the form of “omental pancake,” mostly as a cystic solid mass. However, some tumors are difficult to identify using imaging and often require pathology and immunohistochemistry to confirm the diagnosis.

In conclusion, this is a rare report of ^18^F-FDG PET/CT findings of the colon. ^18^F-FDG PET/CT is valuable in determining the malignancy of PEComas.

## Author contributions

**Conceptualization:** Peiqi Wang.

**Investigation:** Peiqi Wang, Xiao Zhang.

**Writing – original draft:** Qianqian Chen, Xiao Zhang.

**Writing – review & editing:** Jinhe Zhang.
